# CHILDHOOD TRAUMA AMONG US VETERANS AND THEIR NEGATIVE HEALTH AND RISKY BEHAVIORS IN LATER LIFE

**DOI:** 10.1093/geroni/igad104.2578

**Published:** 2023-12-21

**Authors:** Jessika Dayrit, Shirley Salom-Bail, Cesz Islaya, Eunice Castro

**Affiliations:** Department of Veterans Affairs, Menlo Park, California, United States; Department of Veterans Affairs, Menlo Park, California, United States; Department of Veterans Affairs, Menlo Park, California, United States; Department of Veterans Affairs, Agana Heights, Guam, United States

## Abstract

**Background:**

Behavioral and health outcomes of childhood traumas are public health issues. Research on adverse childhood experiences (ACEs) have been explored among the general population; however, ACEs among persons with history of military service remains unknown.

**Methods:**

Current study used secondary dataset obtained from CDC Behavioral Risk Factor Surveillance System (BRFSS) 2011. Study’s total sample size was 7,112. Data was weighted to account for the complex sampling design and nonresponse. Logistic regression was used to explore ACEs among US Veterans, and how early trauma correlates to long-term health problems, while controlling for their socioeconomic status.

**Results:**

Our analyses suggested that compared to participants with 0 ACEs, those who reported 1 ACE (OR=1.38; CI=1.10-1.73), 2 ACEs (OR=2.30; CI=1.80-2.95), 3 ACEs (OR=2.81; CI=2.11-3.73), and 4+ ACEs (OR=4.04; CI=3.20-5.09) were more likely to report depression. Participants who reported for having 4+ ACEs were 4.16 times more likely to report in risky sexual behaviors (OR=4.16; CI= 1.83-9.46). Individuals with 4+ ACEs scores were 30% more likely to report binge drinking (OR=1.30; CI=1.02-1.67), 94% at risk for being a smoker (OR=1.94; CI=1.54-2.45) and had higher odds of developing poor health status (OR=1.67; CI=1.32-2.10).

**Conclusion:**

Our research suggests that more ACEs score associates to a higher risk of developing health issues in later life. Research on ACEs has traditionally focused on health outcomes using secondary datasets among the general population. More research should be explored in longitudinal studies on ACEs, particularly with persons who have served in the military.

